# A Review of the Retinal Impact of Traumatic Brain Injury and Alzheimer’s Disease: Exploring Inflammasome Complexes and Nerve Fiber Layer Alterations

**DOI:** 10.7759/cureus.67093

**Published:** 2024-08-17

**Authors:** Tapabrata Khan, Tamilanban Thamaraikani, Chitra Vellapandian

**Affiliations:** 1 Pharmacology, SRM College of Pharmacy, SRM Institute of Science and Technology (SRMIST), Chennai, IND; 2 Pharmacy/Pharmacology, SRM College of Pharmacy, SRM Institute of Science and Technology (SRMIST), Chengalpattu, IND

**Keywords:** il-18 & il-1β, asc speck, neuroinflammation, lipid metabolism, retinal nerve fiber layer, amyloid beta plaques, nlrp3 inflammasome, retinal neurons, alzheimer’s disease, traumatic brain injury

## Abstract

A huge number of new cases - around a few million of traumatic brain injury (TBI) - are recorded globally each year, making it a major public health risk. A significant portion of all accident-related deaths are attributable to TBI, a notable mortality rate. There are TBI deaths in every age range. Long-term neurobehavioral impacts, such as altered emotions and personalities, cognitive and mental deficits, and so on, are experienced by the majority of survivors. Our main objective is to understand the possible mechanism of the NLRP3 inflammasome in retinal neurons and enhance precision regarding reducing the burden of retinal neurodegeneration in TBI-induced AD. Both primary and secondary insults initiate the intricate pathophysiology of traumatic brain injury. Primary injuries are caused by mechanical force and occur right after the collision. Long-lasting and delayed secondary injuries follow. Studies demonstrating the continuous nature of research on the relationship between retinal neurons and TBI-induced Alzheimer’s disease (AD) include neurodegeneration, retinal changes, and inflammatory response biomarkers. TBI can cause changes that resemble those seen in AD. This includes the accumulation of tau tangles and amyloid-beta plaques, which are also observed in the retina and imply a potential relationship between AD, traumatic brain injury, and retinal health. The linkage between TBI and AD, the effect of the innate immune system in post-TBI AD, the function of immunological moderators, the activation and assembly of inflammasomes in TBI, the pathophysiology of TBI, and the connection between TBI and inflammasome activity were the main topics of discussion in the following discussions. Of particular interest was the potential mechanism by which the NLRP3 inflammasome, in conjunction with SREBP2 and SCAP inflammasome, in retinal neurons in TBI-induced AD. The thinning of RNFL, poor lipid metabolism, and new developments such as drug delivery technologies, lipid metabolism modulation in retinal neurons, and drug-targeting lipid pathways and their mechanisms are then covered in this article.

## Introduction and background

Fifty million new cases of traumatic brain injury (TBI) are reported globally each year, making it a grave public health concern. TBI accounts for 30-40% of all injury-related deaths, which is a significant mortality rate. TBI deaths occur among all age groups. Most survivors experience long-term neurobehavioral aftereffects, including emotional and personality alterations, cognitive and psychiatric impairments, and so forth. These consequences are costly for patients, their families, and society. Both primary and secondary insults bring on the complicated process of TBI pathogenesis [1]. Mechanical force causes the primary injury, which happens immediately after the accident and is followed by a prolonged and delayed secondary injury. Numerous pathogenic pathways can result in secondary damage, including excitotoxicity, oxidative stress, brain metabolic dysfunction, cerebrovascular pathology, chronic inflammatory events, and mitochondrial dysfunction.

**Figure 1:**
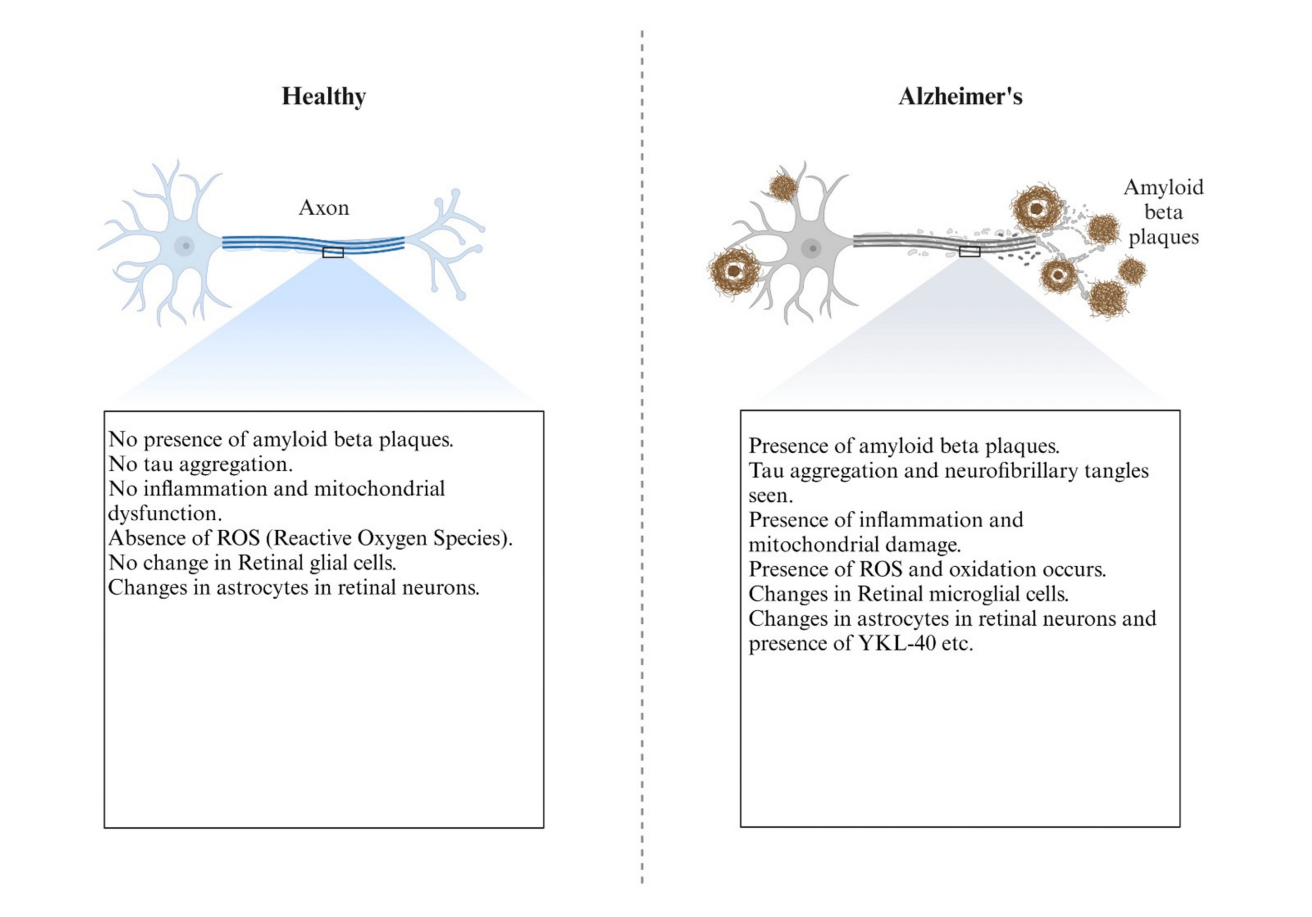
Comparison of biomarkers between healthy and TBI-induced AD neurons I designed this figure on my own (permission is required to use it).

A higher incidence of Alzheimer's disease (AD) and other types of dementia has been linked to traumatic brain injury [2]. Research on the connection between retinal neurons and TBI-induced AD is ongoing, as evidenced by several studies [3]. Similar to the alterations shown in AD, TBI can result in neurodegeneration. This includes the buildup of tau tangles and amyloid-beta plaques (as shown in Figure 1), which are also seen in the retina and suggest a possible connection between retinal health, AD, and traumatic brain injury.

After a TBI, the retina may change. For instance, the retinal nerve fiber layer (RNFL) may get thinner, and the retinal ganglion cells (RGCs) may disappear [1]. These alterations resemble ADs, indicating that TBI may hasten or replicate the retinal neurodegeneration linked to AD. TBI can cause the brain to become inflamed, which could potentially harm the retina. This is known as the inflammatory response. Following TBI, studies have revealed an increase in activated microglial cells and caspase-3-positive cells in the retina, which suggests inflammation and cell death. Biomarkers present in the hippocampus, where the majority of AD forms originate, may be linked to the retina's role in AD. The RNFL thinning brought on by RGC loss is thought to be a possible diagnostic sign for AD and may also apply to AD brought on by traumatic brain injury. The relationship between TBI and AD is being studied using emerging model systems. These models aid in our understanding of the processes by which tTB) causes neuronal dysfunction, AD-related disease, and cognitive decline, which may also impact retinal neurons [2]. According to a few studies, TBI can cause or worsen AD-like pathology, including in the retina. The retina may be a window into the health of the brain and a potential biomarker for the early diagnosis of neurodegenerative illnesses. Alzheimer's disease (AD), the most common type of dementia, is typified by progressive memory loss, cognitive impairment, and alterations in personality. But as of right now, there are no proven ways to stop, prevent, or reverse AD. Alzheimer's disease (AD) is characterized by two primary neuropathological features: extracellular deposits of amyloid beta (Aβ) in the form of senile plaques and intracellular accumulation of neurofibrillary tangles composed of hyperphosphorylated tau. The filaments that make up these tangles are closely spaced and incredibly intractable. The β- and γ-secretases break the amyloid precursor protein (APP) sequentially to yield the approximately 40 amino acid Aβ peptide. Alzheimer's disease (AD) is a progressive neurological disorder primarily affecting memory and cognitive function. This is the most common kind of dementia, characterized by the accumulation of Aβ plaques and hyperphosphorylated tau protein in the brain to form neurofibrillary tangles.

The US Centers for Disease Control and Prevention (CDC) state that persons with a history of moderate TBI are 2.3 times more likely to develop AD, which they attribute to the persistent nature of neuroinflammation that follows TBI [1]. There are numerous other clinical traits shared by TBI and AD, but the most notable is the chronic neuroinflammatory response, which is partly mediated by the innate immune system's ongoing activation of inflammasomes.

The inflammasome is a multiprotein complex that, when caspase-1 is activated, triggers interleukin (IL)-1β and IL-18, which are proinflammatory cytokines that induce pyroptosis, a process that results in cell death. Numerous recent investigations on TBI have shown increased inflammasome activity following injury, mainly in activated microglia. When Aβ buildup occurs in the CNS, microglia are activated, which causes the release of IL-1β in AD. Moreover, Aβ monomers attaching to components of inflammasomes cause Aβ plaques to develop. Recent research shows a connection between the secretion of components of inflammasomes from microglia and the production and accumulation of tau. Together, these findings suggest that targeting the interruption of inflammasome activation may assist in attenuating AD pathology and that the inflammasome may be involved in some of the main pathomechanisms of AD.

In this review, we discuss the pathophysiology of TBI, the inflammasome activity in retinal neurons during TBI-induced AD, and relevant clinical and experimental findings regarding post-TBI inflammatory response and disease on retinal neurons. As an extension of the central nervous system (CNS), the retina is easily accessible, making retinal imaging a very promising tool for initial AD detection. Some applications include (1) optical coherence tomography (OCT), which is the technique used to quantify microvascular alterations and neurodegeneration in the retina. It can identify abnormalities in the microvascular network and thinning of the RNFL, which are suggestive of AD, moderate cognitive impairment (MCI), and even preclinical phases of the disease. (2) The non-invasive technique known as "digital retinal photography" makes it possible to examine the vascular and neuronal structures of the retina. The risk of AD and cognitive function impairment is linked to both qualitative and quantitative data obtained from this imaging. (3) Hyperspectral imaging: This method looks for signs of Alzheimer's disease by analyzing a broad range of light. It describes changes in the retina's light dispersion, which could point to early-stage biochemical alterations in AD. (4) OCT angiography (OCTA) scanning: This technique has modified the retinas of patients exhibiting biological symptoms of AD by mapping the blood vessels in the retina. The retinas of those without AD markers were normal [3].

Retinal imaging has improved as a possible tool for quick, early AD detection and screening because of these imaging techniques and the creation of computer algorithms for picture processing. Finding retinal biomarkers that correspond with brain pathology aims to enable early, non-invasive, and affordable diagnosis as well as tracking the course of the disease. Retinal imaging can help patients by detecting Alzheimer's disease (AD) early and providing various advantages, such as (1) early intervention, which means timely interventions that could halt the progression of the illness are made possible by early detection of AD. This could involve medication, cognitive therapies, and alterations in lifestyle. (2) Improved treatment results: Patients who receive an early diagnosis may be able to start therapies earlier in the course of the illness, which may increase their effectiveness. This may prolong the onset of more severe symptoms and enhance the quality of life. (3) Decreased costs: When compared to other diagnostic techniques like MRI or PET scans, non-invasive retinal imaging is less costly. By postponing the need for more intense treatment, early detection of advanced AD can help save on total healthcare expenses. (4) Planning and support: With a better grasp of the disease trajectory, patients and their families can make plans about care requirements, housing arrangements, and financial arrangements. (5) Research opportunities: It is now feasible to participate in clinical trials for novel therapeutics, which advances our understanding of AD and opens the door to possible future treatments. (6) Psychological benefits: Having early diagnosis information can provide one with a sense of control and the chance to make wise decisions regarding their life and health. All things considered, retinal imaging as a method for early AD identification can provide patients with information and choices, which may enhance their prognosis and quality of life [2].

Some evidence suggests that retinal imaging may be a non-invasive biomarker for the early diagnosis of AD, given the presence of tau pathology and Aβ plaques, two characteristics that are characteristic of AD. The paper underscores that transmembrane aspartic protease, known as β-secretase, and b-site APP cleaving enzyme (BACE) are the rate-limiting enzymes for the generation of Aβ. It is responsible for the initial cleavage of APP to generate a membrane-bound C-terminal fragment. γ-secretase then rapidly cleaves this fragment to generate Aβ because the Ab peptide is heterogeneous, and γ-secretase is a licentious protease. Of all the numerous types of Aβ isoforms, the more hydrophobic Aβ42 and the more soluble Aβ40 make up the majority of the accumulated Aβ in senile plaques. On the other hand, the phosphoprotein tau is the main neuronal microtubule-associated protein that has a preferential axonal distribution. Tau aids in the stabilization and promotion of microtubule assembly, which helps control intracellular trafficking. The level of phosphorylation of tau is a strong regulator of its microtubule assembly activity. The tissue responsible for light detection in our eyes, the retina, is what gives us both image and non-image vision. It is an essential part of the central nervous system. The retina's intrinsically photosensitive ganglion cells (ipRGCs), rods, and cones are examples of photoreceptors that can absorb light signals and translate them into electrical signals. Bipolar cells downstream receive the signals generated in the rods and cones, which are subsequently relayed to RGCs. RGCs then send information to particular brain areas connected to vision via the optic nerve.

**Figure 2:**
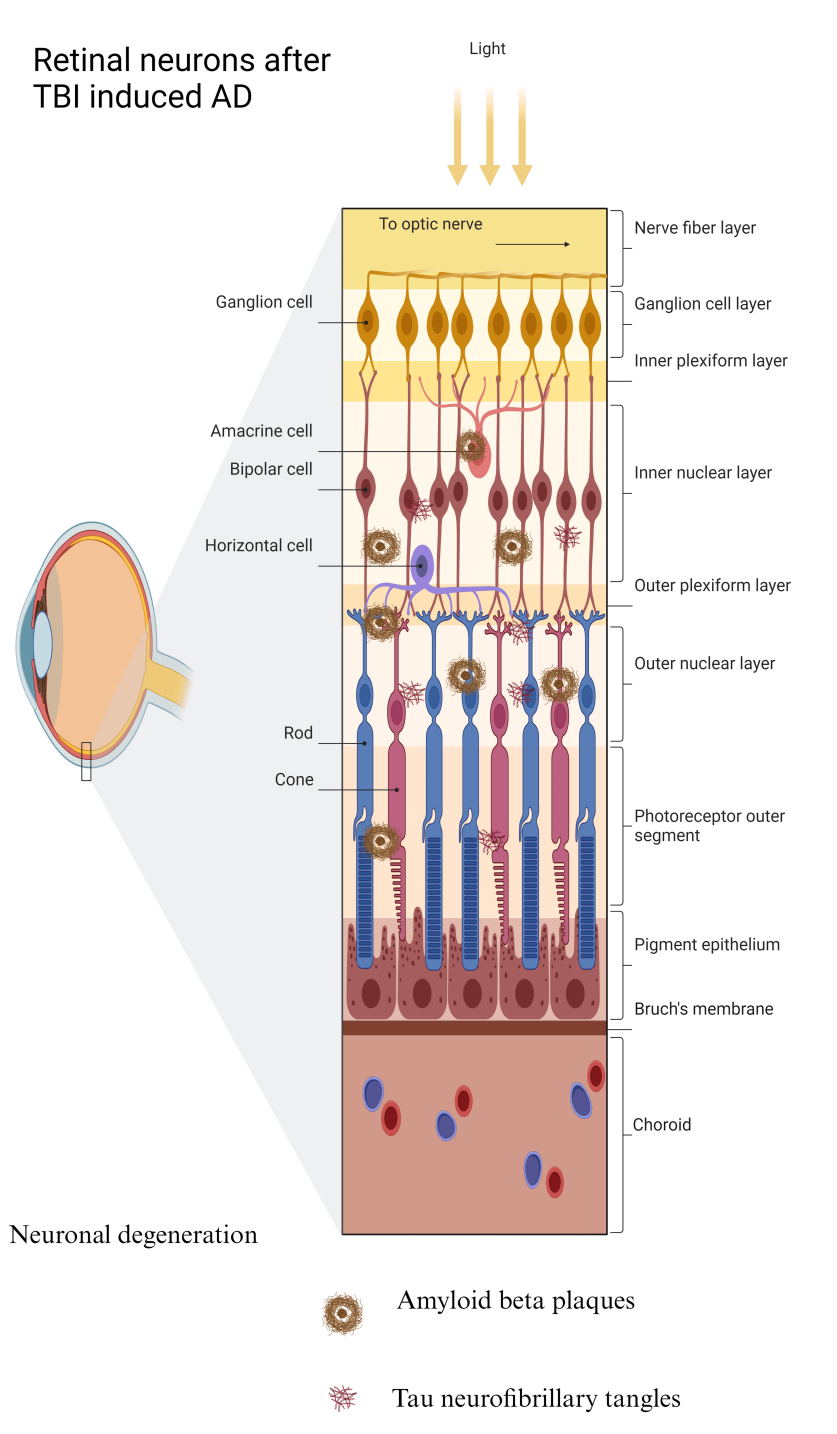
Alterations in TBI-induced AD retinal neurons TBI: traumatic brain injury; AD: Alzheimer's disease. I designed this figure on my own (permission is required to use it).

We also talk about TBI as a causing factor for the onset of AD and the molecular interaction between these two disorders through inflammasome activity. In conclusion, we go over recent translational research and possible treatment avenues for the creation of medications that specifically target the activation of inflammatory cytokines in the retina in the diseases of AD and TBI. Remarkably, there is growing recognition that the retina, an outgrowth of the central nervous system, may mirror the degenerative alterations brought on by AD in the brain. This is because the retina and the brain have numerous physical and functional similarities. Early in the course of AD, retinal neurons may show signs of malfunction. According to studies, people with AD may have changes in their retinas, such as vascular characteristics, destruction of retinal ganglion cells, and weakening of the retinal nerve fiber layer [4]. These alterations are believed to be a reflection of the synaptic disconnections and neuronal death that occur in AD patients' brains. To identify these retinal alterations, researchers are investigating a range of imaging modalities (as shown in Figure 2). This could facilitate an early diagnosis of AD, potentially even before the manifestation of notable cognitive symptoms. Investigating retinal neurons concerning AD is an exciting field of study that could lead to early diagnosis using retinal biomarkers and provide insights into the course of the illness.

## Review

The interplay between TBI and AD

Similar to traumatic brain injury (TBI), AD is a chronic neurodegenerative condition characterized by structural impairment, neuronal loss, persistent inflammation, and aberrant behavior. Because trauma may act as a pathological facilitator for the start of AD in the brains of those who are predisposed to the disease, understanding the commonalities and potential interactions between these two illnesses is crucial. This is particularly relevant since research indicates that traumatic brain injury (TBI) may cause AD pathology to manifest 4-10 years earlier than it would otherwise. Considering that the pathogenesis of AD is characterized by comparable disruptions to cellular homeostasis, microglia, astrocyte activation, and inflammatory activity, the consequences of trauma on the CNS are intriguing [5]. For instance, inflammatory cytokine release, pyroptosis, chronic microglia activation, neuronal damage, NLRP1, NLRP3, AIM2, and ASC speck activity have all been linked to TBI in numerous research and experimental paradigms.

Similarly, comparable findings have been noted in AD pathology and are believed to play a role in its advancement. Aβ is thought to occur in approximately 30% of TBI patients, according to human observations and studies on TBI that have demonstrated increased levels of AD-associated proteins after damage. Furthermore, in mouse AD models, it has been shown that TBI-induced abnormalities in CNS lymphatic functioning result in an increase in microglia and a decrease in the clearance of tau and Aβ. In mouse model investigations of AD using the 3XTg model, elevated Aβ levels have been seen within 24 hours of TBI; it should be noted, though, that these levels often returned to sham levels seven days later. Researching using the 3XTg AD mouse model, a study reported impaired spatial memory function and elevated Aβ concentrations in the hippocampal regions of mice with injuries 28 days after the injury [3]. Comparing tg-ArcSwe AD animals to undamaged AD mice at 12 and 24 weeks after injury, they found indications of earlier development of Aβ pathology as well as impaired learning and memory function in a more chronic injury paradigm. A group of scientists investigated tissue loss and ongoing inflammatory activity following traumatic brain injury (TBI) in the AD mouse model named R1.40 at 3 and 120 days after the injury. They found elevated amounts of Aβ in the brains of three-month-old mice with injuries in the AD mouse model named APP/PS1. When compared to undamaged controls 30 days after injury, the mice hurt when they were six months old and had a lower overall Aβ load but not a fibrillar Aβ burden. Interestingly, in a subclinical blast damage paradigm with 20-week-old mice of the APP/PS1 model, the opposite effects were observed. In this model, recurrent blasts administered three times a week for eight weeks did not lower the total plaque load but did lower the levels of oligomerized Aβ and enhance behavioral outcomes [4].

As much as one-third of Tau pathology has been demonstrated in TBI patients following both once-at-a-time and repeated traumas. It is interesting to note that soldiers of the Vietnam War with traumatic brain injuries (TBIs) who did not have elevated Aβ expression had higher pTau loads in their CSF, according to research carried out by Clark and associates for the US Department of Defense. Following traumatic brain injury (TBI), pTau is also observed in mice and is phagocytosed by microglia with the ability to activate them [6]. From the beginning, one day following the damage, Xu et al. reported increased pTau aggregation following TBI in the mouse model called the P301S model [1,4]. Six months later, they observed impaired learning and memory performance concomitant with increased pTau aggregation (as shown in Table 1). Furthermore, studies have indicated that enhanced plaque formation occurs in the vicinity of sections of the BBB disrupted by aging, making disruption of the BBB resulting from TBI interesting. Taking everything into account, TBI is thought to be a possible initiator of AD occurrence due to the initiation of the death of neuronal bodies, diffuse axonal damage, interference of the blood-brain barrier (BBB), activation of microglia, and inflammasome complex activation, all of which are important factors influencing AD pathogenesis and ongoing research subjects.

**Table 1:** Outcomes after TBI in different AD models Reference citation [3,4]. 3xTg: One of the most used AD models is the triple transgenic (3xTg) mouse, which replicates both the tau and Aβ pathologies; Tg-ArcSwe: transgenic mice with the Arctic and Swedish Alzheimer mutations; R1.40, APP/PS1, P301S: these are commonly used mouse models in Alzheimer's Disease.

AD models	Outcomes	Duration after TBI
3XTg	Elevated Aβ levels, sham Aβ concentrations, raised Aβ levels and lower spatial memory	1 day, 7 days, 28 days
Tg-ArcSwe	Decreased learning along with memory	12 weeks
R1.40	Elevated tissue loss, lifted inflammation	3 days, 120 days
APP/PS1	Raised Aβ levels and reduction in spatial memory	30 days
P301S	Increased tau aggregation, developed tau aggregation, and worsened learning and memory	1 day, 1 week, 6 months

Impact of innate immune system in post-TBI Alzheimer’s disease

Numerous intricate pathophysiological mechanisms underlie trauma. Physical trauma frequently results in cell death, structural damage, inflammation, edema, and infection. These effects either modify homeostasis in more chronic pathologies or are temporary aspects of the initial trauma and healing. The consequences of brain damage (TBI) are not temporary; rather, they cause long-term changes in the context of the CNS, such as modifications of structure and a continuous rise in inflammation [5]. Several biological mechanisms are involved regarding both short-term and long-term illnesses, including the innate immune response. From an evolutionary perspective, all kinds of intricate cell life have retained the native immune system, which is among the earliest lines of defense against invasive species and cellular harm. Along with physical barriers like skin and epithelia, the innate immune system is composed of cellular agents like neutrophils and macrophages. Pattern recognition receptors (PRR), which are genetically programmed, are used by the innate immune system to identify harmful bacteria, undesirable foreign material, and cellular damage. PRRs assist the innate immune system in identifying pathogen- and damage-associated molecular patterns (PAMPs). Examples of PRRs include nucleotide oligomerizing domain (NOD)-like receptors (NLR) and toll-like receptors (TLR). As a result of these patterns, macrophages rapidly adapt to an inflammatory and dynamic phenotype that mediates intruder phagocytosis and the release of inflammatory signaling proteins to alert neighboring cells to potential danger [1]. Similar to this, additional cells have PRR expression and can identify DAMPs and PAMPs, which activate the native immunity to response and cause inflammasome activity in oligodendrocytes, astrocytes, neuronal cells, and microglia [3].

Role of immunological moderators in post-TBI Alzheimer’s disease

It has long been believed that the CNS environment possesses special immunity [5]. A large number of antigen-presenting cells, the BBB, and a variety of anti-inflammatory regulators are involved in the brain's immunological reaction, that control inflammatory processes to a great degree. Recent research has revealed that the system of meninges lymphatics, which lies within the brain's central nervous system boundaries, is the site of a wide range of neuroimmune interactions. Dysfunction in this system exacerbates the diseases associated with traumatic brain injury. Among the various pathways, microglia became identified to be integral players in the central nervous system's consequence of trauma. It is interesting to note that these cells support both neuronal homeostasis and plasticity in addition to serving as first responders to CNS diseases and injuries [7]. When in a "resting" condition, microglia are recognized morphologically within the optimal CNS context and are sampled via lengthy procedures that search for harmful alterations in homeostasis. While in their resting state, microglia also eliminate waste from cells and support the central nervous system's overall plasticity by regulating neurogenesis, maintaining synaptic connections, and pruning off synapses. Following trauma, microglia's TLRs and NLRs identify PAMPs and DAMPs generated by damaged cells. They then promptly enter a stage known as "activation," wherein they travel in the path towards the damage and assume a more ameboid configuration. Microglia are essential for preserving the wellness of the central nervous system by helping to eliminate infections and injured neurons [5].

Furthermore, in response to cognitive injury, they communicate with astrocytes and neuronal cells and are important players in the subsequent pathomechanisms of a range of CNS illnesses. The CNS's well-known source of inflammatory activation, microglia, is mostly controlled by the formation and inflammasome activation. Traditionally, Two variants of stimulated microglia were identified. The M2 form reduces the inflammation-associated activity of the M1 form and is protective of neurons. It also emits neurotropic elements and cytokines that suppress inflammation. The M1 type generates pro-inflammatory cytokines as well as chemokines and is inflammation-promoting. Even though both forms are complementary, research has revealed that the M1 form is more prevalent in chronic inflammatory activity following injury, whereas the M2 form is transient. Recent studies have revealed that there may be other and intermediate kinds of microglia, indicating that the conventional morphological categories of microglia are not exhaustive. The M1/M2 microglial taxonomy is oversimplified, according to studies examining microglia activation following CNS sickness or injury, since multifunctional and other various forms are being observed following a trauma. The regenerative and anti-inflammatory M2 form, for instance, can be further divided into subtypes M2a, M2b, M2c, and M2d. The subtype named M2a, which has been brought up via IL-13 and IL-4, enhances scavenger receptors and promotes phagocytosis, IL-10 production, tissue development, and repair. It is involved in anti-parasitic responses. The M2b subtype, when stimulated by IL-1R ligands, can produce forms in between that display pro- or anti-inflammatory activity by secreting IL-1β, TNF-α, and IL-10. M2b is similar to M1 microglia in that it regulates the M2-mediated response and engages utilizing B lymphocytes. Since it encourages tissue cleansing when inflammatory activity is stopped, the M2c form of inflammation is thought to be resolved. It is activated by glucocorticoids and IL-10. The M2d form is distinct from the M1 form in that it is anti-inflammatory and angiogenic, and it is alternately activated by IL-6 and adenosine receptors.

Recent research suggests that microglia categorization may be more precisely defined along a dynamic spectrum of inflammation action, going beyond the conventional M1/M2 classifications. Instead, microglia should be defined by various parameters, such as the expression of genes or the detection of numerous exterior biomarkers. New categories are particularly significant in AD pathogenesis, even if much current research still refers to anti-inflammatory or pro-inflammatory microglia informally using the M1/M2 categorization. It is believed that a novel type of disease-associated microglia (DAM) that expresses many genes linked to AD is essential to the progression of AD. In response to CNS damage, microglia engage in interactions with neurons and astrocytes [8]. Inflammasome assembly regulates the expression of inflammatory cytokines, which is modulated by microglia and astrocytes while also preserving CNS homeostasis. Astrocytes, the most prevalent cell type in the brain, collaborate with neuronal cells to regulate blood circulation, sustain the blood-brain barrier, control ionic as well as water balances, and alter the exchange of synaptic information. It is interesting to note that astrocytes and microglia share extensive procedures that search their surroundings for variations in homeostasis. With the help of their special end feet, astrocytes cling to neurons and construct intricate networks of gap junctions. These blood vessels comprise the BBB [4]. Through ionic buffering, calcium signaling, glutamatergic and GABAergic neurotransmission, and crosstalk with neurons, astrocytes preserve homeostasis. Following an injury or infection, astrocytes undergo activation (astrogliosis), which is demonstrated by a rise in the cytoskeletal protein named glial fibrillary acidic protein (GFAP). Astrocytes, like microglia, release a variety of inflammation-causing substances, among which are growth factors, reactive oxygen species, and cytokines and chemokines like IL-1β and TNF-α. While several elements might cause astrogliosis, including damaged cell DAMPs, microglia's cytokines, neurons' Aβ, synaptic glutamate, albumin through BBB disintegration, and ATP are known to cause stimulation of astrocytes, the exact mechanism is yet unclear [9].

Inflammasome activation and assembly in TBI

**Figure 3:**
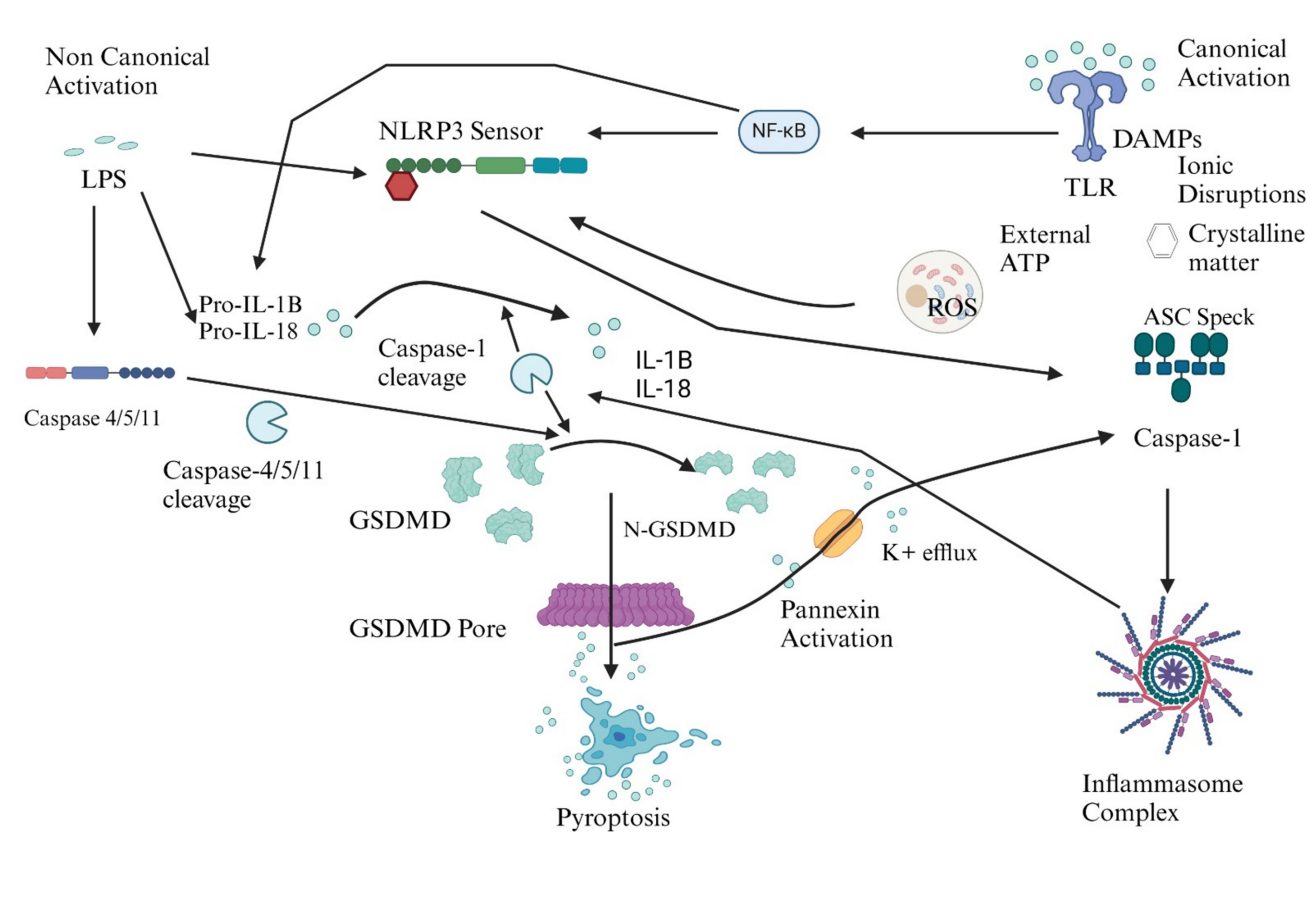
Inflammasome activation and formation in post-TBI AD LPS: lipopolysaccharides; NLRP3 sensor: NOD-like receptor protein 3 sensor; NF-κB: nuclear factor kappa B; DAMPs: damage-associated molecular patterns; TLR: toll-like receptor; ASC Speck: apoptosis-associated speck-like protein; External ATP: external adenosine triphosphate; ROS: reactive oxygen species; GSDMD: Gasdermin D; IL: interleukins. I designed this figure on my own (permission is required to use it).

A multi-protein complex called the inflammasome is created in the course of the innate immune reaction. The sensor protein that identifies inflammatory microbes is known to contain either pyrin, an AIM2-like receptor (ALR), or an NLR. The NLR class of inflammatory mediators is further categorized by the pyrin (NLRP) or caspase (NLRC) stimulation and mobilization protein found in their nucleotide-binding regions, which comprise NLRC4, NLRP3, and NLRP1. Along with caspase-1, adaptor protein apoptosis-associated speck-like protein has a caspase recruitment domain (ASC) and is frequently found in the inflammasome complex [8]. Procaspase-1 can bind and become activated after inflammasome activation thanks to the oligomerization of ASC. Activation of caspase-1 results in the cleavage and activation of IL-18 and IL-1β, and Gasdermin-D (GSDMD), respectively, forming the pyroptotic pore. The outcome is pyroptotic cell death and the extracellular space is filled with inflammatory cytokines and other elements of the intercellular environment (as shown in Figure 3).

The trigger of the corresponding sensor is unique in that it activates the inflammasome. The NLRP1 sensor is activated by microbially related substances like the lethal B. anthrax neurotoxin, which causes alterations to the amounts of double-stranded RNA and ATP in cells. It possesses a CARD, or C-terminus caspase recruitment domain, and a function-to-find motif. Caspases are bound by NLRP1, either directly or via the adaptor ASC [10]. The NLRP3 sensor is special because it can be triggered by two different pathways and a variety of stimuli. Conventional activation occurs via the canonical pathway in response to several stimuli, such as extracellular ATP, DAMPS, PAMPS, intracellular calcium elevations, dysfunctional mitochondria, and potassium efflux in cells. By using caspase-11 in the model of mice or the caspase enzyme-4/5 in human individuals, lipopolysaccharide (LPS) triggers the alternate mechanism of activation, also referred to as the noncanonical pathway. Upon recognition of LPS, GSDMD is cleaved by caspase, causing the pyroptotic hole to develop. This leads to the release of ATP and potassium efflux, ultimately activating NLRP3. The NLRC4 sensor is indirectly activated by protein complexes that bind the enzyme caspase-1 to the CARD directly or indirectly through the ASC. Examples of these proteins are flagellin and needle proteins, which are bacterial [8]. Staphylococcus aureus and other Gram-positive bacteria activate NLRP6, which is being demonstrated to combine with caspase-1, ASC, and caspase-11 to result in an inflammasome complex [11].

AIM2 is categorized by the pyrin domain at its N-terminal and the hematopoietic interferon-inducible nuclear protein at its C-terminus, which has a domain with 200 amino acid repetitions and is not part of the NLR group and needs ASC for binding the caspase enzyme-1. AIM2 is activated by the DNA with the double strands found in the cytosol. Along with the pyrin, which becomes active by inactivating RhoA/Ras homolog family member A, it also possesses two B-boxes, a domain with a coiled coil, and a B30.2 domain at the C-terminus. The primary function of inflammasome activation, independent of the specific trigger or inflammasome in question, is to lessen infection by causing the afflicted cell to undergo pyroptotic cell death and to notify other cells of possible danger [7].

The function of ASC speck in TBI

ASC is an inflammasome component that serves as the scaffold for the inflammasome complex. ASC is structurally comprised of the CARD and pyrin domains, which, in turn, facilitate the interaction of caspase-1 and the sensor proteins associated with the inflammasome. It is expressed by the PYCARD gene. Upon activation of the inflammasome, an ASC speck generated by ASC self-oligomerization and an accumulation of 1 μm can be found. Pro-caspase-1 binding occurs concurrently with ASC speck formation, intensifying the synthesis of cytokines that trigger inflammation and the activity of inflammation. It was discovered by Edwards et al. that instead of blocking the stimulation of the inflammasome or sequential expression by suppressing intercellular ASC speck production dosed with nigericin using colchicine, higher concentrations of nigericin were necessary to trigger the inflammatory response [12]. These results imply that activation of the inflammasome might occur without the need for ASC, as it raises the efficiency of the inflammasome activity by lowering the stimulus intensity required to initiate an inflammatory reaction. That characteristic makes it possible to react more forcefully and quickly to inflammasome stimuli. Extracellularly released ASC particles intensify the inflamed response. Activated microglia within the CNS absorb ASC particles and release cytokines mediated by inflammasomes. As a result, extracellular ASC particles spread inflammation when stimulating microglia, which absorb them and release inflammatory cytokines after their release by pyroptotic cells [10,13].

Additionally, ASC particles can initiate and sustain inflammation upon absorption through adjacent cells and recognition via TLRs. Newer research conducted by Cyr along with others demonstrates that the development of the aging-related inflammation phenomenon is linked to increased production of the ASC protein in the cerebral tissue [9,14]. Similarly, within four hours of brain injury, in the mouse lung tissue, Kerr et al. found enhanced production of proteins named ASC as well as speck oligomerization, which was connected to lung dysfunction following neural trauma. Additionally, Johnson et al. found that blood samples of serum from patients with diabetic kidney disease and lupus nephritis showed elevated production of ASC protein. They also demonstrated how the levels of ASC protein were an effective biomarker to predict the corresponding pathologic consequences, while greater quantities were associated with greater unfavorable consequences [8,15]. Johnson et al. observed a similar pattern within the CNS in the serum from blood taken from hospitalized individuals with brain injuries; ASC along with other proteins associated with the inflammasome were similarly elevated after injury [8,15]. Moreover, patients with the condition called multiple sclerosis showed elevated levels of the ASC and the enzyme caspase-1, which Keane et al. noted as possible pathological indicators. Additionally, Kokiko-Cochran and Godbout also found that ischemic stroke patients' thrombolytic cores had higher concentrations of proteins linked to ASC [13]. Last but not least, Venegas and Heneka demonstrated that patients suffering from AD as well as moderate cognitive impairment reported elevated levels of ASC protein in the serum of the blood. Moreover, those levels additionally constitute a trustworthy indicator of AD pathology's initial stages. Both the ASC protein and the inflammatory complex appear to be necessary when combined in the development of numerous CNS illnesses and injuries, such as AD and TBI [5].

The pathophysiology of TBI

TBI encompasses a wide range of CNS injuries. These include penetrative trauma from gunshot wounds, explosion-related damage from pressure, concussions from sports, and trauma involving auto accidents. There are different degrees of severity for every type of TBI. It is customary to think of a traumatic brain injury as a two-part occurrence. The greater long-lasting harm caused by trauma is referred to as a secondary injury, whereas its early effects are called a primary injury [14]. Primary injury refers to the harm that results from a physical impact on the brain. It can have both localized and diffuse consequences, and it usually happens quickly. Blunt force trauma results in acute cell loss at the lesion's epicenter, as well as possible vascular damage, lymphatic/glymphatic malfunction, edema, and diffuse damage to neural networks and axons. PAMPS and DAMPS, which are important in secondary injury, get released during primary harm from injured and dead cells.

During a secondary injury, various pathological processes triggered throughout the initial damage cause chronic impairment of CNS equilibrium and mental functioning. Ionic equilibrium is upset at the cellular level, leading to energy deficits, malfunctioning microglia, and the synthesis of ROS. After a moderate or more severe TBI, neuroexcitatory glutamate is released as a result of abnormal depolarization from injured neurons. Worse pathophysiological outcomes are linked to higher glutamate release, which also raises intracellular sodium levels and stimulates the synaptic bouton to release glutamate, triggering the release of potassium ions post-synaptically [7]. When calcium inflow meets potassium outflow, external potassium and sodium-potassium channel function increases during an attempt to regain electrolytic homeostasis. The ensuing pump activity uses up cellular ATP reserves, which could be exhausted and not replenished when mitochondria try to store the significant intracellular calcium influx. This malfunction leads to hyperglycolysis and lactate accumulation. The cell goes through the enzymatic pathways required to cause cell death when there is an increase in calcium and a reduction in ATP. In a strategy to treat the damage, microglia produce neurotrophic factors, boost the action of phagocytes, and apply enhanced inflammation to eliminate and fix injured cells and structures. Microglia get activated when wounded, or cells with necrosis develop PAMPs and DAMPs. DAMPs cause astrocytic scarring, which is a tightly packed cluster of astrocytic cells that migrate to the site of injury and emit cytokines that cause inflammation [8].

Furthermore, a lesion that damages the BBB allows monocytes coming from outside the central nervous system to enter, thus intensifying the body's general inflammation reaction. By way of direct blood infiltration into the brain tissue, which causes red blood cells enriched in iron to produce oxidative damage and excitotoxicity, as well as the arrival of immunological cells from elsewhere, which leads to heightened stress from oxidative damage, inflammatory conditions, and cell death, TBI pathogenesis is further exacerbated by circulation inflammation and BBB impairment [7]. Lastly, it is believed that impairment of the lymphatic and glymphatic systems lessens the excretion of neurotoxic proteins from the central nervous system following injury, aggravating inflammatory pathology, edema, and cellular death. Despite being associated with traumatic injury situations, these effects frequently last months or years after the injury, with chronic inflammation increasing the likelihood of neurodegeneration and the emergence of concomitant diseases. Research has demonstrated that impairment to the CNS from stroke or brain injury due to trauma is linked to lung damage as well as cognitive loss, sleep disturbances, cardiovascular problems, and disturbances in the functioning of the gastrointestinal tract. Moreover, genetic susceptibility has been linked to both initial and subsequent damage consequences, even though TBI is primarily a disorder inflicted by an external source [5].

For instance, participants in recent cumulative research, including US military personnel with mild TBI who tested confirmed for a protein called ApoE4, an AD-associated protein, reported worsening psychological well-being along with cognitive performance at higher counts. In addition, it has been demonstrated that, following TBI, like ApoE, rats show an increase in the microglial gene expression linked with AD known as triggering receptor expressed on myeloid cells 2 (TREM2), which may be connected to TBI neuropathology. Such findings imply how genetic predisposition plays a significant influence on TBI outcomes, but they also highlight the individual variability of pathological outcomes, contributing to the tremendous heterogeneity of the TBI population [9].

The link between TBI and inflammasome activity

The chronic inflammatory response following TBI is caused by modifications to the outside of the cell milieu, the distribution of PAMPs and DAMPs, the breakdown of the blood-brain barrier, and the stimulation of microglia. The NLRP3 inflammasome may develop as a reaction to PAMPS, DAMPS, elevated intracellular calcium level, potassium flux, malfunction of mitochondria, and ATP present extracellularly. Through nuclear factor-κB (NF-kB) signaling, TLRs recognize DAMPs produced by damaged cells and enhance the NLRP3 sensor and IL-1β RNA transcription. Furthermore, the inflammasome is activated by modifications within the cell, such as ROS and potassium efflux, as well as ionic alterations like Cl−. The NLRP3 inflammasome has been demonstrated to be a significant factor in TBI pathophysiology, but it is not the only inflammasome that supports the native immune inflammasome response following TBI. For instance, after a traumatic brain injury, the NLRP1 and AIM2 inflammasomes are also triggered [15].

After TBI, the blood and cerebrospinal fluid (CSF) contain released inflammasome proteins. These inflammasome proteins in circulation are reliable indicators for assessing the extent of the injury and the likely pathological consequences that may arise [6]. For example, ASC, caspase-1, and NLRP1 were found in greater concentrations in the CSF of patients experiencing both moderate and severe TBI. These values matched the Glasgow Evaluation Scale ratings for the patients during five months, and higher amounts of protein were associated with worsening endpoints [9]. According to research by Kerr et al., blood serum samples taken from human TBI patients one and two days after the injury revealed raised levels of caspase-1 and ASC and higher levels of ASC were linked to worsening pathological outcomes [4]. The findings by Perez-Barcena et al. are corroborated by their discovery that the measurement of caspase-1 levels in blood serum 24 hours following a TBI hospitalization can reliably forecast clinical results six months afterward [14]. Higher levels of caspase-1 are linked to more severe injuries and significant pathogenic consequences. In an earlier investigation, it was found that individuals with traumatic brain injury (TBI) who had higher intracranial pressure had higher caspase-1 levels in their CSF and that these raised levels were linked to worsening pathological outcomes [13]. Furthermore, Johnson et al. found that levels of IL-13 may be utilized to gauge the harshness of an injury and that elevated caspase-1 and IL-10 in TBI patients' blood serum taken between one and twelve hours after the injury were linked to worsening outcomes [8].

Furthermore, Lee et al. demonstrated in a TBI murine model that at 1 and 2 days following exposure injury, the harmed cerebral cortex's tissue had significantly higher levels of NLRP3, ASC, caspase-1, and IL-1β. ASC speck oligomerization and elevated GSDMD expression followed this [4]. These findings show that following trauma, the NLRP3 inflammasome was not only triggered but also led to an increase in pyroptotic activity and the consequent release of cytokines. While microglial activation and a stream of defense cells into the injured tissue occur, followed by a TBI, the bulk of PAMP and DAMP production post-injury is seen during the initial hours to minutes after damage, often between the initial few days up to a week. The hypothesis that ASC levels increase with microglia activation lends credence to the fact that microglia stimulate inflammasome activity following injury. TBI research that looks at the efficacy of pharmacological inhibition and replacement to lower microglia activity has found a subsequent decrease in brain damage and inflammatory activity. Even though the first week following an injury is when primary and secondary injuries have the greatest negative effects, persistently harmful inflammation lasts far longer than the customary recovery time. Patients with TBI have been shown to exhibit personality abnormalities, generalized mental health problems, and a persistent loss of learning and memory function. Pathophysiologically, inflammation, autoimmunity, and persistently activated microglia can still be found for months to years after the initial lesion. As a result, TBI is thought to be a hazardous factor for several CNS diseases, including disorders like AD that resemble dementia [12].

Possible mechanism of the inflammasome in Retinal neurons in TBI-induced AD

Alzheimer's disease, TBI, and other neurodegenerative illnesses have been associated with inflammatory processes, namely those related to the NLRP3 inflammasome. Activated microglia are the main source of elevated inflammasome activity in TBI, and this can exacerbate AD pathogenesis. This implies that one potential target for improving AD pathogenesis could be inflammasome activity. A growing body of scientific evidence suggests AD is indicated by phosphorylated tau (pTau) and Aβ. Extracellular Aβ deposition has been identified in the retina and is linked to retinal aging and degeneration. Structures such as Bruch's membrane, which has elevated amounts of Aβ, have been suggested as a model for AMD [16]. Phosphorylated tau accumulations have the potential to cause dysfunctions in the release of neurotransmitters and synaptic loss, as well as anomalies in signaling cascades, axonal transport, immunological response, and neurotransmission. Increased immunoreactivity for markers like MCP-1 and F4/80, as well as TUNEL-positive profiles in the RGC layer, are signs that these deposits may be the source of neurodegeneration in the retina. Some evidence suggests that AD is linked to retinal neuronal degeneration.

Retinal deterioration and visual impairments are common in patients with AD and mild cognitive impairment (MCI), which can be linked to retinal neuronal degeneration. Reduced thickness of the layers of the retina, including the RNFL, optic nerve degeneration, and neuronal loss, are among the structural abnormalities of the retina in AD. In the retinas of AD patients and animal models, amyloid plaque deposition, microglial activation, and cell death are observed, which is correlated with the loss of the hippocampus spine. Retinal biomarkers may be used as surrogate biomarkers of early AD since these alterations in the retina can mirror brain abnormalities associated with AD in vivo. In the context of TBI, retinal biomarkers in post-TBI AD are being investigated for their potential to act as markers of the illness's progression. AD pathogenesis may be influenced by the multiprotein complex known as the inflammasome, which is involved in inflammation. Signature reticular signs of AD include tau protein-based neurofibrillary tangles and Aβ plaques, which may be correlated with inflammasome activity [4]. These biomarkers point to the potential for the development of quantifiable retinal biomarkers for the diagnosis of AD in its early stages, together with retinal abnormalities such as aberrant microvascular flow and RNFL thinning. Non-invasive imaging techniques like OCT and OCTA are employed to learn more about these possible biomarkers. Retinal imaging is being investigated as a possible biomarker for AD early diagnosis, and there is a lot of interest in this field.

The use of technologies such as OCT and OCTA to quantify microvascular alterations, neurodegeneration, and retinal architecture is being investigated. These studies seek to enhance non-invasive methods for early identification and monitoring of AD. When retinal cells, including neurons, are activated, the NLRP3 inflammasome can cause the release of inflammatory cytokines such as interleukin-18 (IL-18) and interleukin-1β (IL-1β), as well as pyroptosis, a kind of cell death that results in the release of a significant number of inflammatory cytokines. Retinal disorders may progress more quickly as a result of this mechanism. Complex in nature, the activation of the NLRP3 inflammasome may include multiple biological pathways, including the optimum assembly of the inflammasome through the creation of a ternary complex with sterol regulatory element-binding protein 2 (SREBP2) and SCAP. Moreover, activation of the NLRP3 inflammasome might result from the instability of lysosomes in retinal pigment epithelial cells, which can aggravate conditions like age-related macular degeneration (AMD). Cytokines implicated in inflammatory reactions, IL-18 and IL-1β, have been examined for AD [13]. Similar to IL-1β, IL-18 can control neuronal excitability and prevent long-term potentiation (LTP), a type of neuronal plasticity that is thought to be the basis for memory and learning. Both the latter, the protein synthesis-dependent phase, and the early, p38 mitogen-activated protein kinase-dependent phase are affected by this suppression. Additionally, because neuroinflammatory processes are interrelated, IL-18 can set off a detrimental cycle of inflammation that may also impact the brain and retinal neurons.

Research indicates that the brain cortex of AD models may exhibit altered activation of SREBP2, which may also affect retinal neurons. SREBP2 has been connected to the control of tau and amyloid-β in AD neurons and plays a role in cholesterol metabolism, which is essential for the health of neurons. Although there are no explicit investigations on the effects of SREBP2 and SCAP on retinal neurons in AD, these proteins' overall involvement in neuronal lipid homeostasis raises the possibility that they may be involved in retinal neuron health in AD. Neuronal lipid homeostasis, which is critical for preserving the well-being and functionality of neurons, particularly those in the retina, depends on SREBP2 activity. Reduced SREBP2 activation in the cerebral cortex of AD mouse models raises the possibility that compromised SREBP2 function plays a role in early-stage neuronal dysfunction in the retina in AD. Although the search results do not provide specific impacts on retinal neurons, it can be assumed that similar mechanisms may be at work because retinal neurons and brain neurons share many properties. Defective SREBP2 activation may cause abnormal lipid metabolism in retinal neurons, which could accelerate the development of AD-related disease in the retina and cause retinal neuron degeneration.

**Figure 4:**
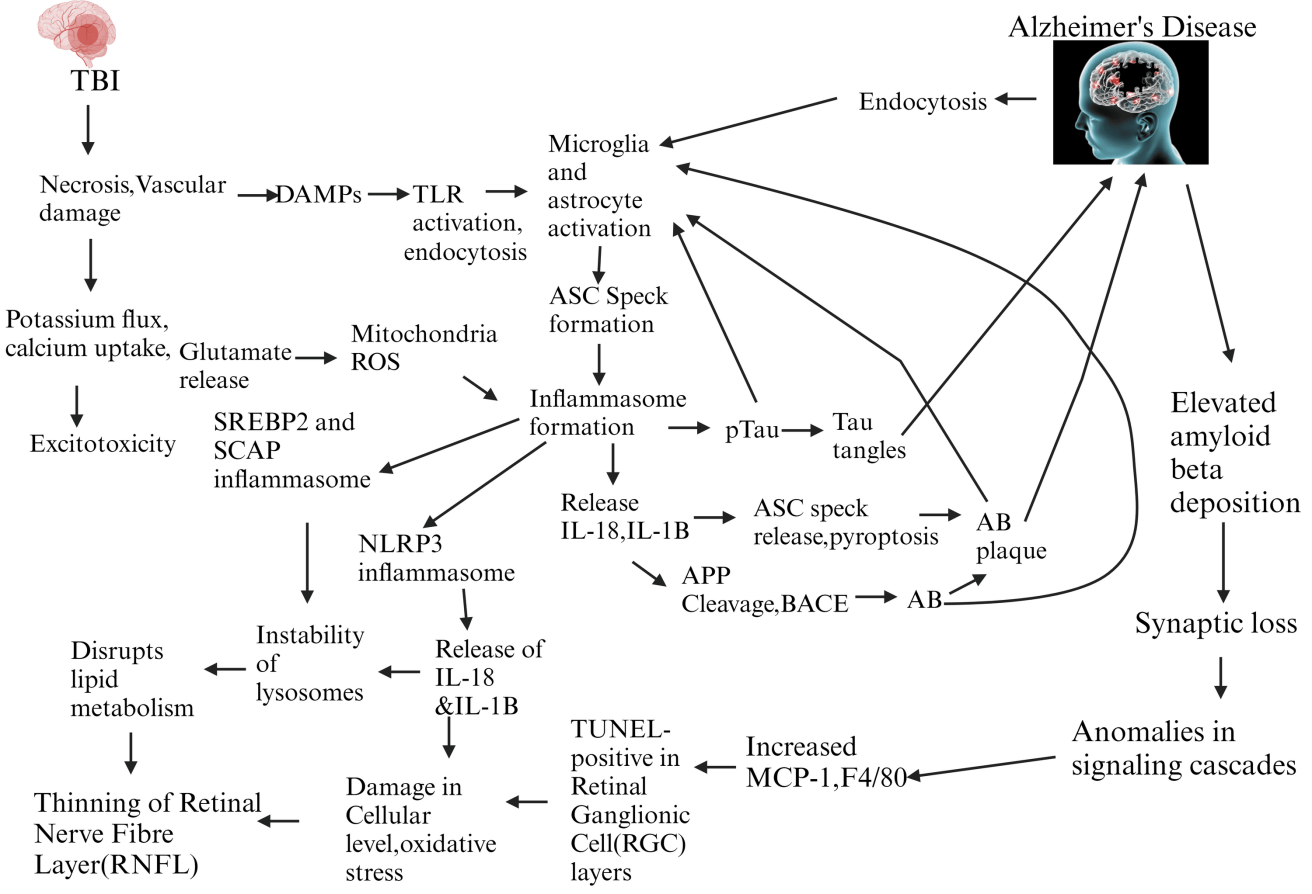
Inflammasome activity in retinal neurons in TBI-induced AD TBI: traumatic brain injury; DAMPs: damage-associated molecular patterns; TLR: toll-like receptors; ASC Speck: apoptosis-associated speck-like protein; ROS: reactive oxygen species; SREBP2: sterol regulatory element-binding protein 2; SCAP: SREBP cleavage-activating protein; IL: interleukin; Aβ: amyloid beta; NLRP3: NOD-like receptor protein 3; APP: amyloid precursor protein; BACE: beta-site amyloid precursor protein cleaving enzyme; MCP-1: monocyte chemoattractant protein 1; TUNEL: terminal deoxynucleotidyl transferase dUTP nick end labeling. I designed this figure on my own (permission is required to use it).

So, in the context of post-TBI AD, after TBI induction, necrosis and vascular damage occur in the brain, and this event leads to potassium flux, calcium uptake, glutamate release, and, on the other side, DAMP activation at the same time. Then, these active DAMPs lead to TLR activation and endocytosis, where the uptake of calcium ions leads to excitotoxicity. Meanwhile, glutamate assists ROS or reactive oxygen species, which are found in mitochondria, in activating the inflammasome complex. Meanwhile, the active TLR triggers microglia and astrocyte activation, which leads to actuated ASC speck formation that drives inflammasome formation, as we discussed earlier. Then the inflammasome complex triggers phosphorylated tau to form tau neurofibrillary tangles and is also accountable for the activation of microglia and astrocytes. These tau tangles are mainly responsible for the occurrence of AD. Some evidence suggests that the inflammasome complex also stimulates the SREBP2 and SCAP inflammasomes, as well as the NLRP3 inflammasome. Furthermore, IL-18 and IL-1β are also liberated after inflammasome formation. IL-18 and IL-1β help during the formation of APP and BACE that activate amyloid-beta (Aβ), which redirects microglia and astrocyte activation along with the formation of amyloid-beta plaque (Aβ plaque). Diversely, IL-18 and IL-1β release ASC speck and stimulate pyroptosis that actuates the formation of Aβ plaque, which energizes astrocyte and microglia activation and is also considered liable for AD. The active Aβ also stimulates the processes of microglia and astrocyte activation. Now, a growing body of scientific evidence suggests that elevated Aβ deposition is seen in post-TBI AD models. This elevated Aβ deposition causes synaptic loss that results in anomalies in signaling cascades. Then, in retinal neurons, increased levels of MCP-1 and F4/80 are noticed. In adherence, TUNEL-positive retinal ganglionic cell (RGC) layers can be noted. This event results in damage at the cellular level in retinal neurons and increases oxidative stress. On the contrary, NLRP3-inflammasomes IL-18 and IL-1β freed and caused instability of lysosomes in retinal neurons. It also actuates damage at the cellular level and increases oxidative stress. At the same time, SCAP and SREBP2 inflammasomes also cause instability in lysosomes. This disrupts lipid metabolism in retinal neurons. Furthermore, after an increase in oxidative stress and lipid metabolism disruption, the diminishing of the RNFL is detected in the retinal neuronal level of TBI-induced AD by inflammasome activity. The inflammasome complex results from microglia and astrocyte activation following retinal neuron necrosis and vascular damage in traumatic brain injury (as described in detail in Figure 4). Alzheimer's disease is a condition that results from this incidence. This illness is characterized by increased amyloid beta deposition, synapse loss, abnormalities in signaling cascades, increased oxidative stress on cells, and damage to cells. Conversely, inflammasome development triggers the synthesis of IL-18 and IL-1B, which ultimately activate the layer of retinal nerve fibers, which becomes thinner by further stimulating the NLRP3, SREBP2, and SCAP inflammasomes.

Role of lipids in retinal synaptic function

In the retina, lipids are essential for synaptic function because they encourage the growth of synapses, which is glia-derived cholesterol. For instance, crucial for presynaptic differentiation and improves synaptic development in RGCs. Encouraging continual synaptogenesis that provides the stability of neurotransmitters, which are necessary for synaptic transmission, depends on cholesterol, which is also critical for continuing synapse development. Lipids, including glycerophospholipids, sphingolipids, and sterols, actively participate in the endocytosis and exocytosis of synaptic vesicles and are implicated in the dynamics of synaptic membranes [3,4]. These roles highlight the significance of lipids in preserving normal synaptic activity and retinal neural transmission.

Effects of impaired lipid metabolism in retinal neurons

Retinal neurons with impaired lipid metabolism may experience several negative consequences later on, such as bioenergetic insufficiency, which means that to make up for reduced lipid consumption, neurons may rely more on glycolytic products for oxidative phosphorylation. However, this can lead to energy deficiencies and worsen the condition of the neurons. Stress that results in neuronal death is caused by metabolic abnormalities brought on by defective lipid metabolism, which can lower the oxygen and nutritional supply to retinal tissue. Stressors resulting from defective lipid metabolism can upset the protein and metabolic balance inside cells, which, if left unchecked, may cause malfunction and even cell death, which is known as cellular homeostasis disruption [3,5]. These outcomes demonstrate how crucial healthy lipid metabolism is to retinal neurons' survival and ability to operate.

Lipid metabolism in retinal neurons in post-TBI AD

Through a variety of methods, lipid metabolism significantly influences retinal neurons in AD. Cholesterol metabolism and neuroinflammation: AD onset and progression are associated with abnormal cholesterol metabolism, and neuroinflammation can be controlled to reduce the buildup of amyloid-β peptides. Impact on Aβ and Tau pathology: Oxidative stress, mitochondrial malfunction, and compromised synaptic transmission are how lipid metabolism is linked to AD. Additionally, it intensifies neuronal impairment and cognitive deterioration by impacting tau and Aβ pathologies. Apolipoprotein E (APOE) secretion: Certain cells release APOE to supply lipid particles and vitamins to neurons, as well as to cleanse neurons by eliminating potentially hazardous "lipid waste." Reduced lipid metabolism: This reduced metabolism has been linked to neurodegenerative illnesses such as AD, impacting cell communication and other physiological functions within the brain [16]. These results underscore the complex relationship, especially concerning retinal neurons, between lipid metabolism and the pathogenesis of Alzheimer's disease.

Therapeutic strategies target lipid-related pathways to enhance retinal synaptic health in TBI-induced AD.

To improve synaptic health in the retina, therapeutic approaches that focus on lipid-related pathways include the study of regulators of lipid pathways. Research is being conducted to better understand the signaling lipid pathway and create new therapeutic approaches that target this mechanism by looking at upstream regulators of lipid pathways. Inhibiting important enzymes is a possible therapy strategy by using autotaxin inhibitors, which have been demonstrated to restore the excitation state of neuronal networks impacted by hereditary diseases. Lipidomic neurobiology is to increase our understanding of lipid messengers that regulate signaling cascades and support cellular protection, repair, and differentiation in the nervous system, which includes the retina. Multi-target treatment approaches such as drugs that target many pathways, such as the lipid-lowering medication probucol, have the potential to prevent and treat neurodegenerative disorders [17]. These approaches are part of a growing body of research that aims to treat retinal and other neurological problems by restoring synapse integrity and function.

Modulation of lipid metabolism in retinal neurons in post-TBI AD

In AD, modifying lipid metabolism to safeguard retinal neurons may entail the following: controlling γ-secretase activity, where the enzyme controls the metabolism of lipids and connects cholesterol to deficits in synapses. Its activity can be changed to affect gene expression linked to the production of cholesterol in neurons as well as cholesterol levels. Modifying lipid metabolic pathways to suppress the expression of pro-inflammatory genes and enhance the expression of anti-inflammatory genes by targeting dysregulated fatty acid metabolism, glycerophospholipid metabolism, and sphingolipid metabolism. Targeting glial lipid metabolism to increase lipid diffusion from cells in the microglia may diminish pathophysiology identical to AD, indicating that glial lipid metabolism should be the primary target. This suggests that the APOE4 variation may be involved in both neuroinflammation and poor lipid management. Using polar lipids and modulating certain receptors, polar lipids can reduce astrocyte pro-inflammatory signaling and alter AD pathogenesis [18]. Exercise enhances biological processes and molecular pathways linked to metabolic problems in AD, particularly lipid metabolism. These strategies emphasize the possibility of lipid metabolism-based therapeutic intervention to preserve retinal neurons in AD.

Drug delivery systems in the retina for TBI-induced AD

There are multiple approaches to guaranteeing medication delivery to the retina for efficacious treatment. Sustained-release systems such as drug-eluting particles and implants are examples of sustained-release devices that offer a steady release of medication over an extended period. Injections: the most common way to administer medication to the back of the eye is by directly injecting it into the vitreous or the tissue immediately around it. Controlled delivery platforms that include implanted eluting devices and sustained-release platforms. Systems based on nanotechnology may be used in future therapeutics. Topical, systemic, and periocular routes are the additional techniques being investigated for posterior segment delivery, which include intraocular injections and the suprachoroidal region. Technologies used in nanomedicine: the potential for delivering drugs to the retina by nanoparticles, micelles, dendrimers, microneedles, liposomes, and nanowires is being investigated [19]. The goal of these techniques is to retain safety and efficacy while offering retinal therapy that is more lasting.

Recent Strides

Drugs Targeting Lipid Pathways and Inflammasome Complex in Retinal Neurons in Post-TBI AD

Medications targeting inflammasome pathways as a potential treatment for post-TBI AD are used to stop, minimize, or undo the consequences of traumatic brain injury and the pathology of AD. A plethora of research investigations and possible therapeutic strategies are presently under investigation. Enticing therapeutic targets are presented by the pathway of inflammasome activation to lessen harmful inflammation and subsequent damage pathways in both scenarios. These could involve limiting the function of the inflammasome sensor protein, like NLRP3, preventing the oligomerization of ASC specks, focusing on caspase-1 activity, or preventing NF-κB from stopping the beginning phase, or stage 1 [20]. Treatments that target NLRP3 activity, including MCC950, have decreased TBI's IL-1β and caspase-1 activity and boosted Aβ phagocytosis in AD, as well as NLRP3 and microglia activation. These early results are encouraging. Research employing the VX-765 as a caspase-1 inhibitor has shown that, during a TBI murine model, there is a reduction in inflammatory cytokine and GSDM-D activity. In a model of AD mice, the beginning of inflammation is delayed, but there is no change in plaque load. Research utilizing the anti-ASC medication IC100 demonstrates remarkable findings in decreasing inflammation and enhancing relevant results in models of mice with acute lung injury generated by CNS injury, multiple sclerosis, and inflammation. Targeting ASC also decreased the expression of the enzyme caspase in rats that had pyroptotic cell death following traumatic brain injury. ASC is a promising target because of its unique function in the inflammatory process and its connections with Alzheimer's proteins associated with Alzheimer's and trauma disorders. Inhibiting ASC speck activity could slow the progression of plaque, activate the inflammasome less, and lessen inflammatory signaling [11].

Certain medications that target lipid pathways are being researched as potential treatments for post-TBI AD as candidate agents. These medications have potential therapeutic benefits, such as adenosine phosphate, oxidized Photinus luciferin, BMS-488043, bisphenol A, benzo(a)pyrene, and ethinyl estradiol. Issues with the neuronal metabolism of lipids in the early phases of AD are being investigated. Regulation of cholesterol has been determined that the main protein target for preserving cholesterol homeostasis and regulating the expression of enzymes essential for the synthesis of cholesterol is SREBP2. These medications and targets are part of the continuing research efforts (as explained in Table 2) to modulate lipid metabolism and create viable therapies for AD.

**Table 2:** Recent developments targeting inflammasome and lipid pathways in post-TBI AD Reference citations [3,4]. AD: Alzheimer's disease; TBI: traumatic brain injury; mTOR1: mammalian/mechanistic target of rapamycin 1; PPAR: peroxisome proliferator-activated receptor; NFkB: nuclear factor kappa B; AhR: aryl hydrocarbon receptor; Aβ: amyloid beta; NLRP3: NOD-like receptor protein 3; GSDMD: gasdermin D; ASC Speck: apoptosis-associated speck-like protein containing a CARD (caspase recruitment domain); IL: interleukin.

Name of drug	Disease	Target	Ongoing study	Recent outcomes
Adenosine phosphate	AD	mTOR1 pathway	Clinical trial	Reduce oxidative stress and increase regenerative phase
Oxidized photinus luciferin	AD	PPAR signaling pathway	Clinical trial	Reducing IL-1β, reducing inflammation
BMS-488043	AD	NFkB pathway disruption	Clinical trial	Reduce Aβ plaque formation
Bisphenol-A	AD	Modulate lipid metabolism	Preclinical study	Reduce Aβ plaque formation
Benzo(A)pyrene	AD	AhR pathway	Preclinical study	Reduce inflammation
Ethinyl estradiol	AD	Cortical lipid metabolism	Preclinical study	Reduce oxidative stress and Aβ formation
MCC950	TBI, AD	NLRP3	Preclinical study	Increased Aβ phagocytosis, lessened IL-1β and caspase-1 function
VX765	TBI, AD	Caspase enzyme-1	Preclinical study	declined GSDMD and cytokine activity, delayed inflammatory onset
IC-100	TBI	ASC speck activation pathway	Preclinical study	Lowered caspase-1 and pyroptosis

Mechanism of Action for These Lipid-Targeting Drugs

Lipid-targeting medications have a variety of different modes of action when used to treat AD, such as changing lipid metabolism. This strategy focuses on the connections that exist between lipid metabolism and important AD pathogenic pathways such as bioenergetic deficiency, oxidative stress, neuroinflammation, and myelin degradation [12].

Preventing tau phosphorylation and the synthesis of amyloid: Certain medications, such as DHA-hydroxylated derivatives, can alter the lipid composition of membranes and improve the symptoms of Alzheimer's disease.

Reducing neuroinflammation: Lipid efflux transporter expression in the glia is increased by liver X receptor (LXR) agonists, which may aid in lowering neuroinflammatory reactions that encourage neurodegeneration [13]. These medications target brain lipid metabolism and microglial function to address various elements of AD neuropathogenesis.

## Conclusions

In conclusion, there are several processes involved in the complex interaction between retinal health and post-TBI AD, including lipid metabolism, AB deposition, and inflammasome activation. The pathophysiology of TBI and post-TBI AD, as well as primary and secondary injury, mechanical force, excitotoxicity, oxidative stress, metabolic dysfunction, chronic inflammation, mitochondrial dysfunction, and factors linked to AD risk, have all been covered in this article. The relationship between retinal neurons and TBI-induced AD, neurodegeneration, retinal alterations, tau pathology, SREBP2 and lipid metabolism, ASC speck, pyroptosis, elevated MCP-1 and F4/80 levels, and the potential connection between inflammasome activation and RNFL thinning by the NLRP3 inflammasome are all discussed in this article. We talked about several medication delivery methods, including sustained-release, periocular, systemic, topical, nanomedicines, and injections, for retinal health following TBI. Clinical trial drugs include adenosine phosphate, BMS-488043, and bisphenol-A, and their mechanisms include lipid metabolism regulation, reduction of neuroinflammation, and tau and amyloid change. These novel strategies could lead to safer and more efficient retinal treatments for AD. Through comprehending lipid metabolism and microglial activity, we are getting closer to effective therapies for this intricate neurodegenerative illness. Comprehending the mechanism could lead to innovative therapeutic approaches that effectively target NLRP3 inflammasome-related and lipid-related pathways to the retina in the treatment of AD following traumatic brain injury.
